# Preparing the next generation of Complex Networks and Systems scientists: Evaluation results for the Complex Networks and Systems NSF research training program at Indiana University

**DOI:** 10.1371/journal.pone.0334779

**Published:** 2026-01-27

**Authors:** Michael Ginda, Katy Börner, Olga Scrivner, William Trochim, Luis M. Rocha, Selma Šabanović

**Affiliations:** 1 Department of Intelligent Systems Engineering, Luddy School of Informatics, Computing, and Engineering, Indiana University, Bloomington, Indiana, United States of America; 2 Department of Computer Science and Software Engineering, Rose-Hulman Institute of Technology, Terre Haute, Indiana, United States of America; 3 Brooks School of Public Policy, Cornell University, Ithaca, New York, United States of America; 4 School of Systems Science and Industrial Engineering, Binghamton University (SUNY), Binghamton, New York, United States of America; 5 Department of Informatics, Luddy School of Informatics, Computing, and Engineering, Indiana University, Bloomington, Indiana, United States of America; Xi'an Jiaotong University, CHINA

## Abstract

Assessing and evaluating programmatic outcomes of graduate education programs help stakeholders understand and respond to challenges that emerge over the course of a complex academic program. Given the increased complexity of program activities and outcomes, there is a need for semi-automatic, interactive visual analytics tools that transform data into actionable insights to inform decision-making. This paper documents the results of the evaluation planning and annual workflow setup to create dynamic assessment reports for the Complex Networks and Systems NSF Research Traineeship (CNS NRT) program at Indiana University. The CNS NRT evaluation workflow relied on institutional, survey, and publication data and the freely available tools to guide decision making and communicate the achievements of faculty and doctoral trainees who participated in the program between 2017 and 2024. The evaluation data to date show that the program is judged by participants as meeting its goals and that there has been considerable evidence of research productivity as indicated in the publication data. These clear gains in short-term outcomes show that the CNS NRT is on a pathway to achieving the medium and longer-term goals of the project, which will be examined over a longer period.

## Introduction

The NSF Research Traineeship (NRT) program awards fund research-based graduate education that focused on high priority interdisciplinary or convergent research areas using comprehensive, evidence-based training models that develop the skills, knowledge, and competencies of student need to pursue a range of STEM careers, which align with workforce development and research needs. NRT programs often involve strategic collaborations with a wide variety of organizations, encourage participation from groups that are underserved and underrepresented in STEM, and develop institutional capacities in graduate education [1]. Since its inception, NSF has awarded over 250 graduated education programs through the NRT funding mechanism. In 2017, Indiana University was awarded an NRT grant (grant ID: 1735095) to develop a graduate training program in complex networked systems.

### Complex Networks and Systems NRT model

The Complex Networks and Systems NRT (CNS NRT) established a unique interdisciplinary training for thirty-four (34) doctoral students plus twenty-two (22) funded trainees. The program aims to train “bi-disciplinary” scientists and to establish the necessary team culture for solving interdisciplinary problems. The CNS NRT training model combined an integrated dual PhD academic education with hands-on, team-centered research. Additionally, the program proposed training 40 affiliate doctoral and master students from Indiana University who were not required to complete a dual doctoral degree; instead, affiliates students received a stipend to complete summer research projects under the guidance of CNS NRT faculty.

Doctoral fellows enrolled in the NRT worked towards a dual PhD program in both Complex Networks and Systems (CNS) and a partner doctoral program from the cognitive, natural, and social sciences. Doctoral fellows were partnered with faculty advisors from both doctoral degree programs who acted as mentors throughout the students’ time in the program. Faculty advisors helped connect doctoral fellows to research projects that relate to their academic disciplines.

The CNS NRT program focused on four major goals:

1. Dual research proficiency: doctoral fellows would develop proficiency in computational and mathematical training to infer, analyze, and model complex networks and systems; and learn experimental and methodological training from domain-specific natural and social sciences.
2. Collaborative skill development: doctoral fellows would be integrated into problem-driven, interdisciplinary projects with teams of leading scientists who collaborate to study multi-level phenomena and publish research with impact in more than one discipline.
3. Workforce development: the program would recruit, train, and place in top research institutions or companies a cohort of students who thrive in the integrated team culture and can solve problems which simultaneously require proficiency in general methods and deep domain specificity. The program was also designed to increase the participation of underrepresented groups in STEM science.
4. Interdisciplinary training model: the program would support the development of doctoral students into experts who can combine two dimensions of science and establish the necessary team culture that supports research geared towards solving complex, multi-level problems facing society. The challenge of interdisciplinary research is to cross the various fields devoted to each level of organization without trivializing any of them.

To accomplish these goals, faculty developed specific course and exam requirements based on agreements and memoranda of understanding between constituent departments, summer research internships, an annual research showcase, professionalization training, a colloquium series with extended opportunity to interact with speakers, open house, and informal special events.

### Interdisciplinary graduate education program evaluation

The evaluation of interdisciplinary STEM education programs poses unique challenges [2,3]. Unlike more traditional subject oriented education evaluations, interdisciplinary programs are more institutionally and academically complex, needing to address not just single participant but also multi-person outcomes, due to emphasis on teamwork. Evaluation studies of multiple graduate education programs highlight several reported impacts that interdisciplinary training and mentorship programs have had on their participants.

A key outcome for all NRT programs is the development of interdisciplinary research skills and competencies of participating graduate students. Interdisciplinary training evaluation models provide a foundational basis for assessing students’ interdisciplinary learning and acculturation in graduate education programs. Borrego and Newswander [4] applied content analysis to analyze 129 Integrative Graduate Education and Research Traineeship (IGERT) programs and humanities doctoral programs to identify specific learning outcomes associated with interdisciplinary training. They identified five key competencies: disciplinary grounding, integration, teamwork, communication, and critical awareness. This framework has been used for structured evaluation of interdisciplinary training programs and aligns with curriculum design aimed at fostering these competencies [5].

Interdisciplinary competencies are often evaluated using surveys and self-assessment methodologies. August, et al. developed a self-assessment tool for graduate students to evaluate their interest and knowledge of disciplinary and multi-disciplinary research methods and practices [6]. An evaluation of the University of Wisconsin’s IGERT program on biodiversity conservation under novel ecosystems, which surveyed graduate students on interdisciplinary collaborations and their research practices, showed that participants with more collaborations reported higher levels of support within their community and expressed stronger support for interdisciplinary practices [7]. More recently, an evaluation of the Ridge 2 Reef NRT (R2R NRT) program at the University of California employed a reflection-based methodology that asked students to articulate their learning experiences and competencies acquired through interdisciplinary training [8]. Graduate students who participated in the R2R NRT reported significant gains in their perception of their interdisciplinary skills, like communication and leadership. Estaiteyeh, et al. developed a mixed-method approach for the self-assessment of graduate students in a medical program that combined quantitative pre- and post-surveys with qualitative reflections on perceived development across seven competencies associated with interdisciplinarity, including: problem-solving, communication, leadership, critical reflection, working in diverse teams, project management, and decision making [9].

Lastly, programmatic evaluations can also include structured feedback from graduate students and faculty members regarding their collaborative experiences. Doctoral mentoring frameworks allow for assessing the impacts of multi-mentor arrangements, which can facilitate development of skills and competencies that are critical to interdisciplinary work [10]. O’Meara and Culpepper examined how the NRT for doctoral students in the interdisciplinary language sciences at the University of Maryland facilitated meaningful interactions among students and faculty across disciplines, thereby enhancing creativity, innovation, and sense of interdisciplinarity of participant graduate students [11,12].

Interdisciplinary STEM education assessment is thus best approached via multi-faceted, mixed-methods systems thinking [13–16]. Consequently, the evaluation committee designed and implemented a comprehensive evaluation plan for the CNS NRT program that is consistent with widely used evaluation models [17], including STEM evaluation [18,19]. The evaluation plan incorporated program theory [20] and logic [21], qualitative and quantitative mixed methods [16], a variety of stakeholders and participants [22–25], formative and summative designs [26], and nonexperimental, quasi-experimental and randomized experimental designs [27–29]. In the following sections we describe the comprehensive evaluation plan for the CNS NRT.

## Evaluating the CNS NRT

The comprehensive evaluation plan was implemented in stages over the five-year program (2017–2022) plus 1 year no-cost extension. During this time, the CNS NRT Evaluation Committee engaged in an annual assessment of the program implementation and outcomes. Given that some students still need to complete their PhD, we collected data until 2024. The evaluation committee’s work was staged into three major parts:

1. Evaluation Planning (Year 1). Identification of internal evaluation resources; stakeholder analysis and mapping; development of logic model and (causal) pathway model; creating of comprehensive evaluation plan.
2. Evaluation Implementation (Years 2–4). Collect, enter, clean and secure data; explore and summarize data; conduct statistical tests; synthesize and interpret results.
3. Evaluation Utilization (Year 5). Engage stakeholders in reviewing summary of data and analysis results and aid with interpretation; revise program model; utilize findings; disseminate results to stakeholders, the community, and the Web.

During the first year of the CNS NRT, evaluation committee members designed an evaluation model, developed tools, and collected data from NRT fellows and NRT faculty. The Systems Evaluation Protocol (SEP) was used to structure the program models and the evaluation plan [30]. The SEP platform allows for designing a set of comprehensive networked pathways between stakeholders, program activities, and outcomes. These pathways are mapped to data collection and management plans via the Netway, a web-based cyberinfrastructure [31].

### CNS NRT stakeholders

**Fig 1:**
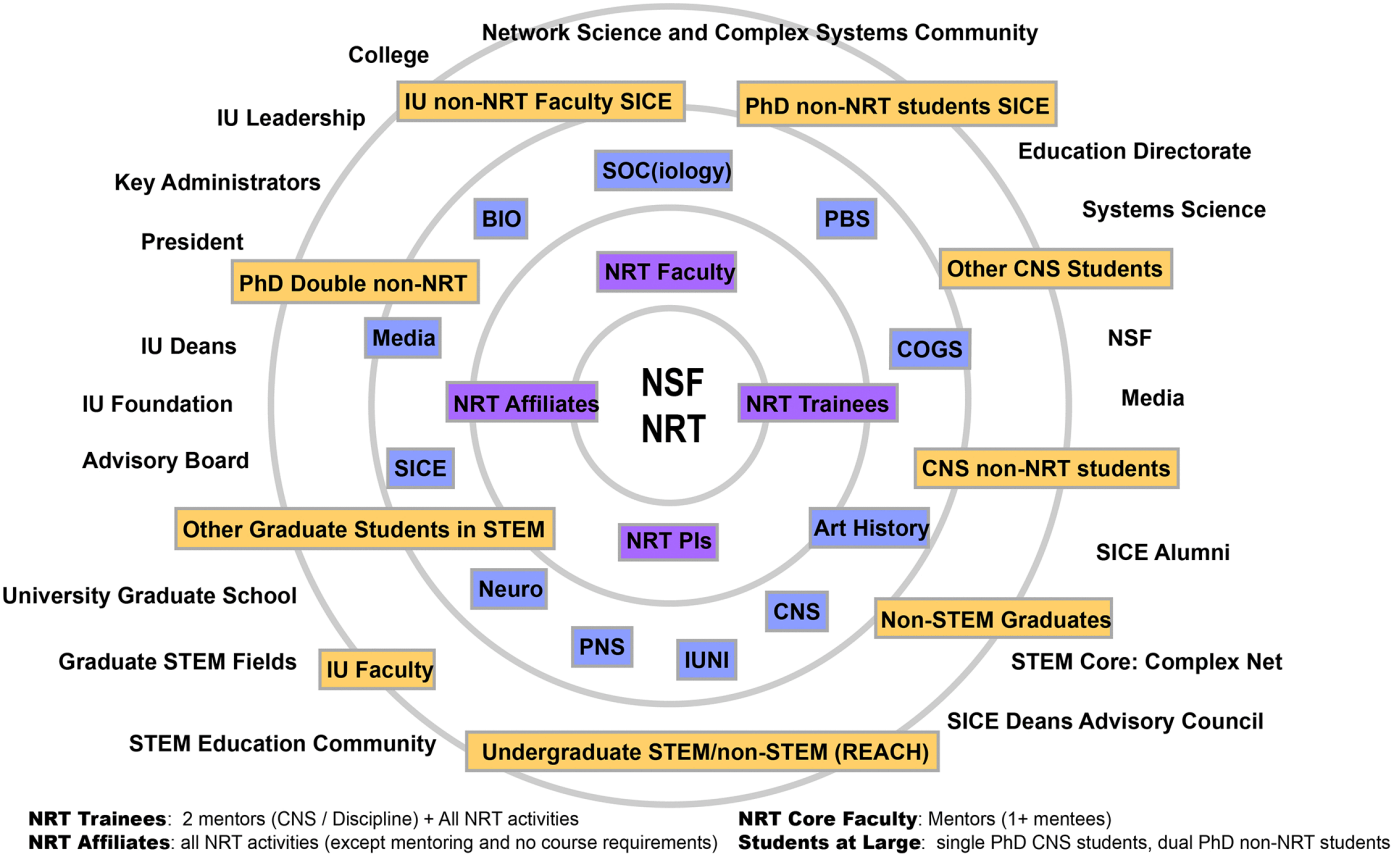
CNS NRT stakeholder model shows the core, proximal, and peripheral stakeholder groups identified by the evaluation team.

As part of the SEP design process, the evaluation committee identified stakeholders with interest in the CNS NRT program, as visualized in Fig 1. The core stakeholders of the NRT include doctoral trainees and affiliate graduate student researchers, NRT faculty advisors and mentors, and NRT administrators supervising the awards. Doctoral fellows admitted to the NRT are enrolled in dual-doctoral degrees, combining CNS and a secondary discipline. They must be involved in all NRT activities and have two mentors/advisors. NRT affiliates are doctoral and master students who participate in summer research experiences and may participate in all non-curricular and advising NRT activities. Core NRT faculty are approved by principal investigators to mentor doctoral fellows and are assigned one or more mentees. The NRT principal investigators manage program administration, budgeting, event planning, committee meetings, and grant reporting efforts. Proximal to the core stakeholders are the affiliated academic departments that host NRT doctoral trainees and faculty, which include the Luddy School of Informatics, Computing, and Engineering, and departments, such as complex networks and systems (CNS), informatics (INFO), cognitive science (COGS), psychology and brain sciences (PBS), neurology (Neuro), sociology (SOC), media and society (MSCH), art history and folklore (ARTH & FOLK), political science (POLS), history and philosophy of science (HPSC), biology (BIO), and physics (PHYS). Last, the stakeholder model identifies peripheral stakeholders at Indiana University and from the public who may be impacted by the work of the NRT.

### CNS NRT activities, outcomes and pathway model

**Fig 2:**
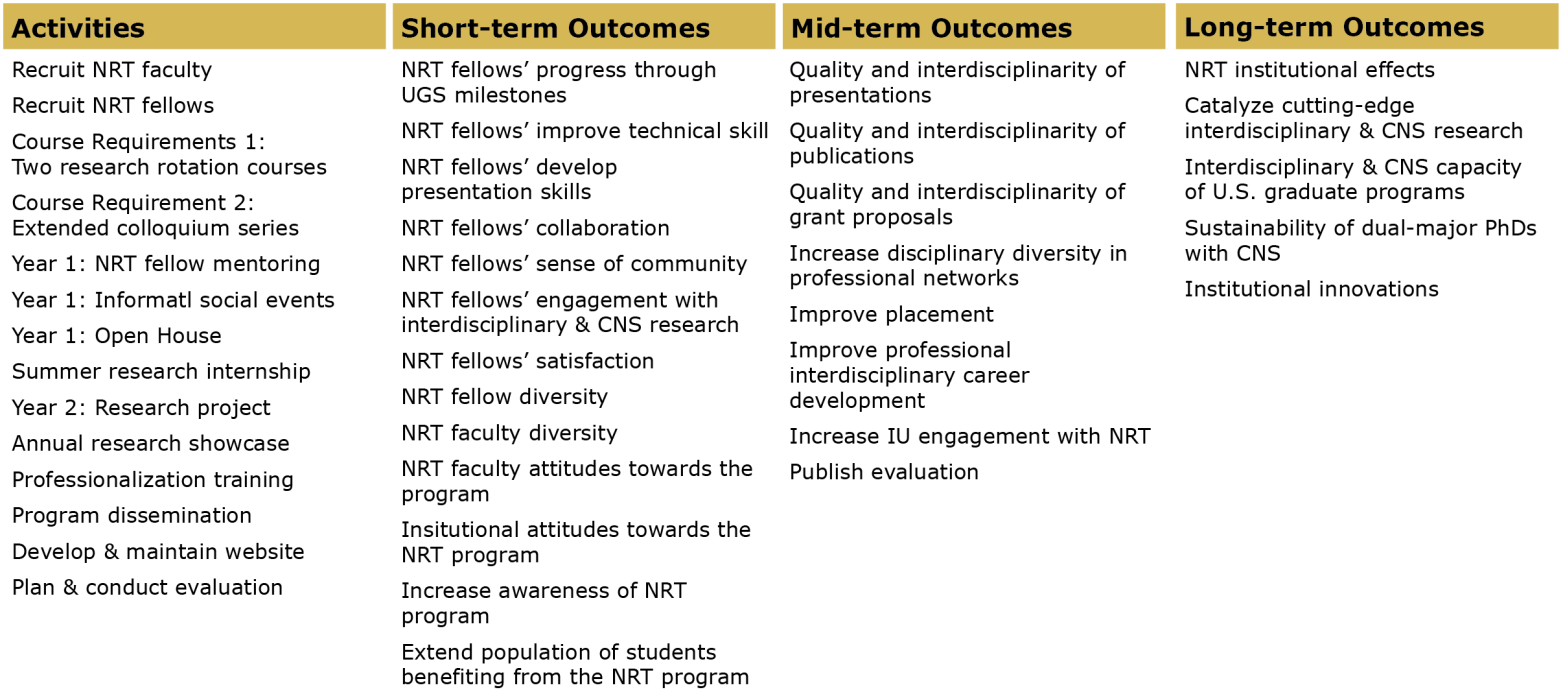
Program activities, short-, mid-, and long-term outcomes identified by the CNS NRT Evaluation Committee.

The CNS Evaluation committee also identified the major activities that stakeholders participated in during their time with the NRT program, which are outlined in Fig 2. Program activities are intended to achieve CNS NRT goals outlined in the grant proposal. Activities fall into four categories: recruitment, curricular design and implementation, training activities, and program evaluation. The CNS NRT evaluation committee identified program outcomes to measure how well program activities and stakeholder participation achieved their goals. Program outcomes are divided into short-, mid-, and long- term objectives in Fig 2. Short-term outcomes tend to focus on NRT doctoral fellows’ academic progress, skill development, and research activities, as well as core stakeholders’ perceptions and satisfaction with the program. Mid-term outcomes focus on the quality of the scholarly research (i.e., presentations, publications, and grant proposals) and professional activities (i.e., professional placements, networking, and career development) of doctoral fellows, and increased engagement with Indiana University.

**Fig 3:**
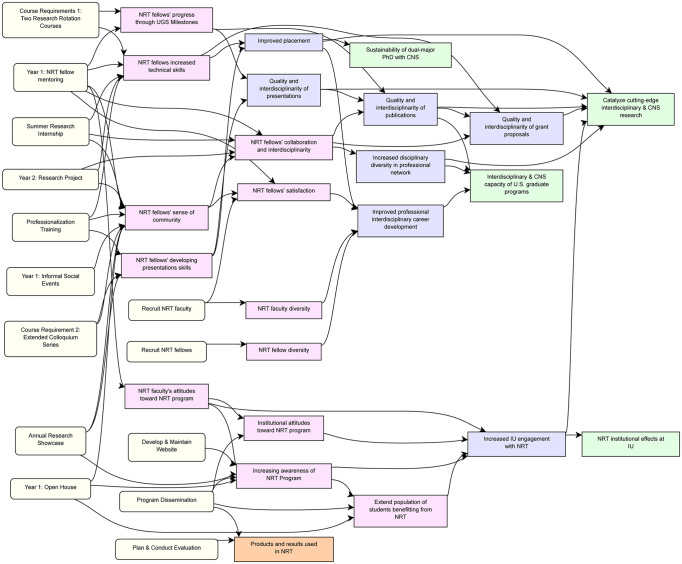
The pathway model connects program activities, with short-, mid-, and long-term outcomes.

The evaluation committee developed a pathway model that connects CNS NRT program activities to programmatic outcomes, as visualized in Fig 3. Each pathway shows a different program activity connected to short-term outcomes. Implicit in this model is that achievement of short-term outcomes has a positive impact on the achievement of later outcomes. The downstream impacts of early successes were a driving motivation for the annual evaluation undertaken by the CNS NRT evaluation committee, which is described in the next section.

### CNS NRT annual evaluations

From 2018 to 2022, the CNS NRT Evaluation Committee engaged in an annual evaluation of the doctoral fellowship program. The goal of the evaluation was two-fold:

1. *Implementation evaluation* efforts assessed the NRT program implementation and provide feedback useful for strengthening implementation efforts.
2. *Outcome evaluation* efforts assessed short-term outcomes for NRT program activities and provide initial reporting on long-term indicators reflective of major goals.

This paper summarizes the three major areas of focus: the program activities, the program short-term outcomes, and the stakeholders’ perceptions and sentiment.

Our evaluation relied on four main data sources for the evaluation, which included: 1, Indiana University’s Student Information Systems (SIS) data that captures student demographics, academic program enrollment, course work, grades, academic advisors, and doctoral academic milestones; 2, CNS NRT administrative membership and participation records for activities, including: annual research symposium, guest speaker talks, skills seminars; 3, CNS NRT annual survey responses from doctoral fellows, faculty, and administrators; 4, publication and conference papers authored by NRT participants and reported to in annual student reports or identified secondarily in publication databases via grant acknowledgements.

## Methods

Student enrollment data was accessed from CNS NRT data archives and annual survey data (2019–2023) was accessed from Qualtrics servers on October 21, 2024, through April 10, 2024, for demographic and survey analysis presented in publication and preparation of supporting data files. The Indiana University Human Research Protection Program approved this study under protocol number 1902712905. The Indiana University HRRP determined that the project does not require IRB review for the following reason: (1) the project does not meet the definition of research as defined by 45 CFR 46.102(d); (2) the project does not meet the definition of research as defined by 45 CFR 46.102(d); and (3) study meets the criteria for approval defined by the HRPP Policy on IRB Review Process.

Consent was not asked of participants in the annual surveys. Survey response data was anonymized by the CNS NRT evaluation managers prior [KB, MG, OS] prior to sharing data with other authors. Student enrollment records were accessible to authors, as they were permitted access to these data as members of the CNS NRT evaluation committee OR as Principal Investigators for the CNS NRT program. Student enrollment records are not shared; instead, information is reported using aggregated statistical data tables.

### Doctoral fellow demographics

Administrative records for the CNS NRT doctoral fellows were collected from Indiana University’s Student Information System, and include students’ gender, race & ethnicity, year of admission, and their secondary academic doctoral program. Administrative records are analyzed to provide statistical descriptions of CNS NRT doctoral fellows overall. Cohort level descriptive statistics are presented to preserve the privacy of the comparably small population of graduates.

### Survey analysis

CNS NRT Evaluation Committee administered an annual survey each year between 2019 and 2023; surveys were conducted July 1–31, 2019; September 10–30, 2020; April 12 to May 31, 2021; April 11 to May 31, 2022, April 20 to May 31, 2023. The committee invited faculty, doctoral students and staff members that participated in CNS NRT program activities during the prior year to measure stakeholder perceptions of the CNS NRT program, its activities, and how well the program has achieved short-term goals. Doctoral fellows were asked to self-assess the development of their research skills, interdisciplinary orientation, and mentorship relationships. Faculty mentors were also asked to assess the quality of their mentoring. The 2021 and 2022 CNS NRT surveys also included questions related to the impact of the COVID 19 pandemic on the program. The survey results were analyzed and presented to the Executive Committee membership to help identify areas for improving program administration and activities based on student feedback. Responses from the 2022 annual survey for doctoral fellows and faculty stakeholders are presented in this paper, as they are representative of the feedback provided across all annual surveys conducted for the program. Survey questions are provided, with annual indices that identify if questions were asked during a given year’s survey, and definitions for the Likert scale used in multiple choice questions are provided in the S1 File.

### Publication analysis

Publication data associated with CNS NRT funding that is reported by doctoral students and faculty provide documentary evidence of successes in training and mentoring doctoral students in research, collaboration, communication, and presentation skills, and, due to the nature of the CNS NRT, interdisciplinarity. Scientometric statistical analysis allows for the assessment of CNS NRT stakeholder (faculty, doctoral fellows, and associate doctoral students) participation, output and impact of research efforts connected to the doctoral program. Publication data enables calculation of annual publication statistics for each author (i.e., year of first CNS NRT publication; overall publication; annual and cumulative publication counts).

Social network analysis (SNA) methods complement scientometric analysis of publication. Author relationship data was extracted from publication and analyzed via co-authorship network analysis to assess collaboration among graduate students, faculty, and other researchers and faculty mentorship efforts and to identify communities of emerging researchers with various network analysis algorithms. Co-authorship network analysis relied on EndNote [32], R language [33], Openrefine tool [34,35] and its suite of lexical clustering algorithms [36], and the igraph library [37] and Gephi (v.0.10.1) [38] for network analysis and visualization. Temporal co-authorship network analysis and visualizations show change over time.

## Results

Data analyses and visualizations provided actionable insights for NRT PIs, faculty, students, and staff to optimize and communicate program demographics and diversity. Details are presented below. A GitHub repository (https://github.com/cns-iu/cns-nrt-evaluation-supporting-information) is used to share the data, scripts used for data preparation, analysis and visualization, and results and visualizations which allow other researchers to review and reuse this work.

### Program demographics and diversity

A total of 35 doctoral students were admitted to the CNS NRT between 2017 and 2021. The 2017 cohort of students also included four students previously admitted to the Indiana University doctoral programs between 2015 and 2016. To date, a total of 7 doctoral fellows (20%) have graduated from the program with dual doctoral degrees; 20 fellows (50.4%) are still working towards their degrees. Additionally, 9 (25.6%) fellows left the program for various reasons. University records indicate that four former NRT students graduated with master’s degrees in informatics; four former NRT students remain enrolled in doctoral or master’s degree programs at Indiana University; last, one student earned a doctoral degree from another institution.

Data for students admitted to the CNS NRT program were managed by the graduate studies office in the Department of Informatics in the student information system used by Indiana University. The SIS includes demographic data on students that the NRT program recruited and admitted between 2017 and 2021. Demographic data includes students’ gender, ethnicity and educational achievements, and their declared secondary doctoral degree program. Demographic data is used to assess how well the CNS NRT met its goals for student diversity over the life of the program.

Demographics for students are self-reported in their applications to the CNS NRT program. Demographic data for students who were recruited to the CNS NRT or who applied and were not admitted are excluded from the statistical analysis. The count of CNS NRT doctoral fellows’ race and ethnicity by the year students were admitted to the program are reported in Table 1. Race and ethnicity counts use a classification implemented by the Informatics Graduate School Office. By the end of the program, 34.3% of admitted doctoral fellows were Asian or Black/African American. Similarly, the count of CNS NRT doctoral fellows’ gender is reported in Table 2. Over the course of the CNS NRT, the female identifying students made up 40% of the admitted students. For two years, there was parity by gender for admitted students.

**Table 1:** CNS NRT doctoral fellow ethnicity & race, by admission year.

Ethnicity	2015-2017	2018	2019	2020	2021	Total	Percent
White	3	4	7	6	3	23	65.7
Asian	2	3	1	3	1	10	28.6
Black/African American	0	1	0	0	0	1	2.9
Not Provided	0	0	0	1	0	1	2.9
Total	5	8	8	10	4	35	100

**Table 2:** CNS NRT doctoral fellow gender, by admission year.

Gender	2015-2017	2018	2019	2020	2021	Total	Percent
Male	4	5	4	5	3	21	60
Female	1	3	4	5	1	14	40
Total	5	8	8	10	4	35	100

All students admitted to the CNS NRT program had either a Bachelor of Arts (BA) or Bachelor of Science (BS), with one student having two bachelor’s degrees. About 23% of the admitted students had been awarded a Master of Arts (MA) or Science (MS). Table 3 shows the reported academic degrees earned prior to admission to the CNS NRT program.

**Table 3:** CNS NRT doctoral fellow education background.

Degree	Students	Percent
Bachelor of Arts	17	48.6
Bachelor of Science	19	54.3
Master of Arts	2	5.7
Master of Science	6	17.1
**Total**	**35**	**100**

The CNS NRT program is designed as a dual-doctoral degree program, where students receive training in interdisciplinary research practices that align across their primary doctoral degree in complex networks and systems and their chosen secondary doctoral program. CNS NRT doctoral fellows participated in 15 different secondary doctoral degrees at Indiana University. Most CNS NRT doctoral fellows chose either cognitive science, informatics, or intelligent systems engineering as their secondary doctoral degree. Table 4 provides an overview of the secondary doctoral programs that CNS NRT fellows selected.

**Table 4:** CNS NRT doctoral fellow secondary doctoral degree.

Affiliation	Count
Cognitive Science (COGS)	12
Informatics (INFO)	5
Intelligent Systems Engineering (ILS)	3
Sociology (SOC)	3
Political Science (POLS)	2
Art History (ARTH)	1
Economics (ECON)	1
Folklore (FOLK)	1
Geography (GEOG)	1
History & Philosophy of Science (HPSC)	1
Media Arts and Sciences (MCSH)	1
Neural Sciences (NEUS)	1
Physics (PYSH)	1
Psychological and Brain Sciences (PBS)	1
Public Health (SPH)	1
**Total**	**35**

#### Faculty demographics.

The NRT was led and administered by five principal investigators for the program and had an additional 29 core-faculty members that serve as instructors and mentor-advisors for doctoral trainees and summer research affiliate graduate students. Core faculty members come from ten disciplines, aside from complex networks and systems. The programs with the highest faculty participation include twelve faculty members from informatics, six faculty from psychology and brain sciences, and three faculty members from biology. Faculty participation by department affiliations are detailed in S2 Table.

### Annual survey

The major component of the CNS NRT evaluation was the annual survey, which was run in April and May of the academic year from 2019 through 2023. The survey invited members of the CNS NRT (i.e., NRT faculty mentors, doctoral fellows, summer research affiliate students, and program administrators) to assess the program based on their experiences over the prior academic year. The core survey contains 68 question-item combinations that were asked each year. The survey was updated each year to add questions that improved the quality of responses or addressed topics of interest to the CNS NRT evaluation and executive committees. For instance, in 2020, the survey added questions for summer research affiliate graduate students; in 2022, the survey added a set of questions asking participants to identify program activities they think should be continued, adapted, or dropped after the program funding concludes.

For this analysis, responses from all five years of the survey were combined to provide an opportunity to understand how CNS NRT participant perceptions of the program changed over time, and how the feedback provided by participants was used by the CNS NRT Executive Committee in decision making and program administration. The analysis follows the major thematic areas covered in the survey. The survey questions are divided into sets, where the first set of questions were asked of all participants; these questions address topics like: participants’ perception that the CNS NRT has met its goals for the year; the impact of COVID-19 on the program (2022–2023); and what aspects of the program should be preserved after the program concludes; the remaining questions are reserved for CNS NRT doctoral fellows, faculty mentors, and summer research affiliate students. Doctoral fellows are asked about their satisfaction with CNS NRT program and associated activities, whether the program impacts their research skills, interdisciplinary skills and self-perception, and about the impact and quality of mentorship relationships they have with faculty. Faculty mentors are asked about their perceptions on the impact mentoring relationships have on their student’s research skills. Affiliate graduate students are asked about their experience in the summer research program.

#### Survey response rates.

Each year, the evaluation committee invited between 45 and 94 individuals associated with the CNS to respond to the annual survey. The average response rate between 2019 and 2023 is 46%; however, after removing responses with low completion rates (<40%), the average response rate for this period drops to 40%. A review of annual data shows that for the first three years, survey response rates ranged between 78% in 2019 to 47% in 2021; however, the final two years of the survey saw significant drop off in participation across all NRT membership groups. The groups least likely to participate in the surveys were affiliate graduate student researchers; however, faculty mentors saw large declines in participation in later years compared to doctoral fellows. Overall annual response rates and rates of responses used in the analysis are presented in Table 5, while annual response rates for different NRT membership groups are shown in Table 6.

**Table 5:** CNS NRT annual survey overall response rates, 2019–2023.

Year	Invitation	Responses	Rate	Responses (Used)	Rate (Used)
2019	45	35	77.8%	35	77.8%
2020	94	53	56.4%	45	47.9%
2021	87	44	50.6%	41	47.1%
2022	87	30	34.5%	25	28.7%
2023	83	21	25.3%	14	16.9%
**2019–2023**	**396**	**183**	**46.2%**	**160**	**40.4%**

**Table 6:** CNS NRT annual survey overall response rates by NRT role, 2019–2023.

NRT Role	Year	Invitation	Responses	Rate	Responses (Used)	Rate (Used)
Faculty	2019	26	18	69%	18	69%
	2020	35	17	49%	14	40%
	2021	31	12	39%	12	39%
	2022	33	9	27%	7	21%
	2023	32	7	22%	6	19%
Doctoral Fellow	2019	12	10	83%	10	83%
	2020	29	22	76%	19	66%
	2021	27	18	67%	18	67%
	2022	31	15	48%	13	42%
	2023	29	9	31%	7	24%
Affiliate	2019	0	0	NA	0	NA
	2020	21	5	24%	5	24%
	2021	21	8	38%	7	33%
	2022	19	3	16%	2	11%
	2023	19	4	21%	1	5%
Admin.	2019	7	7	100%	7	100%
	2020	9	9	100%	7	78%
	2021	8	6	75%	4	50%
	2022	4	3	75%	3	75%
	2023	3	1	33%	0	0%

#### CNS NRT program goals.

In the first section, all participants are asked to: a) rate their level of agreement that the CNS NRT has met its program goals over the past year; b) whether the program has helped disseminate the latest research findings on Complex Networks and Systems, c) whether it increased awareness of Complex Networks and Systems locally, i.e., within Indiana University, and d) whether it increased awareness nationally. Participants indicate their level of agreement using a six level Likert scale, which includes the following values: *Strongly Agree* (6), *Agree* (5), Somewhat *Agree* (4), *Somewhat Disagree* (3), *Disagree* (2), *Strongly Disagree* (1); participants may also select *Not Applicable* (0) if they do not have a response, which are excluded from the analysis. Descriptive statistics are calculated after converting Likert values to a numeric score (i.e., the values in the parenthesis above). Unless indicated otherwise, this Likert scale is used by participants to evaluate their agreement to all questions in this survey.

**Fig 4:**
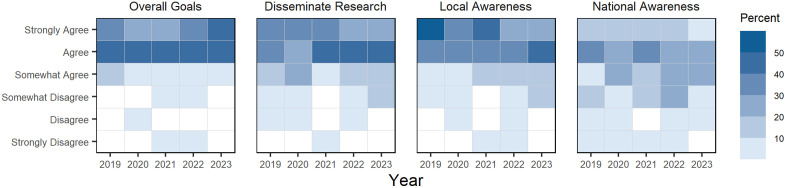
Survey participants’ agreement that the CNS NRT has achieved its program goals, 2019–2023. Each square is shaded to represent the percentage of all survey participants who share the same level of agreement that the CNS NRT program met four major program goals; lighter shades indicate a smaller percent of participants provided an answer, while darker shades indicate a larger percent of participants provided an answer, and white squares indicate no one provided this answer during a year.

Between 2019 and 2023, over half of survey participants agreed that the CNS NRT program had achieved its overall goals for the year; however, there was limited disagreement between 2020 and 2022. Participants generally agree that the CNS program helped with disseminating research in the complex networks and systems, with the strength of agreement shifting from “strongly agree” to concentrating in “agree” over the five-year period. Likewise, there was general agreement over the period that the CNS program helps with local awareness of complex network science; however, there was not strong levels of agreement that the program helped with national awareness. Participants’ level of agreement (percent) to questions about the CNS program are visualized using Likert-heatmaps in Fig 4.

**Fig 5:**
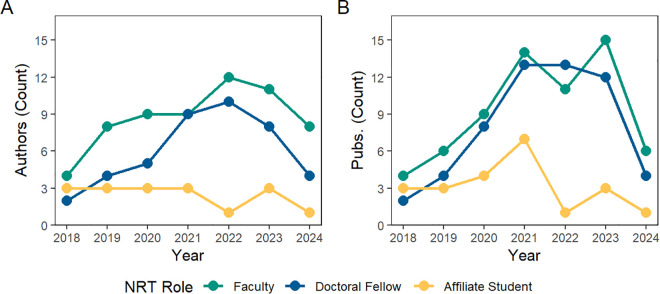
Survey participants’ “level of agreement” that the CNS NRT has achieved its program goals, by NRT role, 2019–2023. Each square is shaded to represent the percentage CNS NRT Faculty, Doctoral Fellows, Summer research affiliates, and program administrators who share the same level of agreement that the CNS NRT program met its four major program goals. Data is provided in S1 Dataset, S1A Table.

We can further break down responses to these four questions by CNS NRT membership groups, which are visualized in Fig 5. When we examine responses by membership groups, it becomes clearer that NRT faculty, affiliates, and administrators tend to provide agreeable responses to questions about whether the CNS NRT program has met its annual goals, helps to disseminate research on and increase local and national awareness of complex networks and systems. Meanwhile, doctoral fellows are more likely to disagree with these questions of whether the program helps disseminate research and increase awareness of complex networks and systems. It is not clear from the survey what drives the difference in responses across CNS NRT membership groups, but there may be key differences in group level perspectives that underlie variation in responses, such as faculty and administrators’ engagement, experience, and knowledge of the university system, research cycles, and disciplinary scholarly communications activities that doctoral students are not yet familiar with from their studies and work.

#### Doctoral fellow assessment of academic progress, research skills & interdisciplinary orientation.

A significant part of the annual survey asks NRT doctoral fellows to assess their satisfaction with CNS NRT program and progress towards their doctoral degrees and to assess the program’s positive impact on their academic research skills and interdisciplinarity (i.e., “interdisciplinary” skills and personal values), or ability to engage in research across/within at least two academic disciplines as scientists. Each question asks doctoral fellows to indicate their level of agreement to statements related to different CNS program academic goals and activities, research skills, and interdisciplinary skills and values. For each of these questions, we analyze and visualize participants’ level of agreement to question items over the survey period.

**Fig 6:**
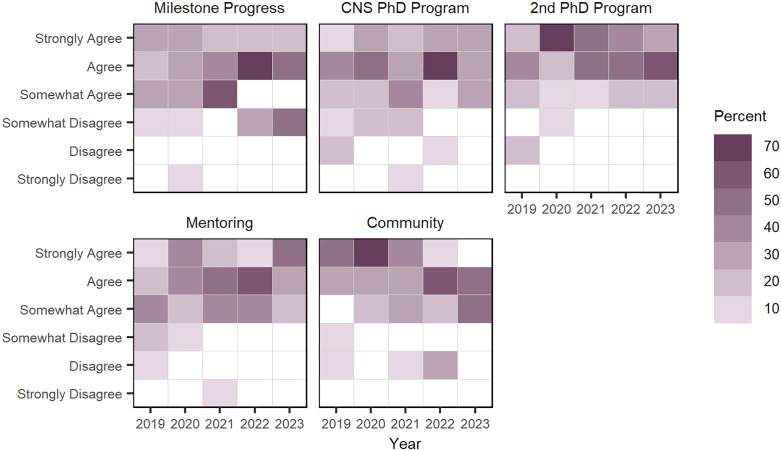
Doctoral fellows’ “level of agreement” that they are satisfied with CNS NRT program and their academic progress, between 2019–2023. Each square is shaded to represent the percentage of doctoral fellows’ level of agreement that they are satisfied with the CNS NRT program and their progress towards five program goals.

Each year, doctoral fellows are asked to indicate their level of agreement that they are satisfied with the CNS NRT program (i.e., mentoring and community) and their progress towards their doctoral degrees (i.e., progress towards doctoral milestones; CNS PhD Program, & 2nd PhD program).Annual responses to doctoral fellow’s agreement that they are satisfied with the CNS NRT program and their academic progress are using Likert-heatmaps in Fig 6; see Table A in S2 File about doctoral fellows’ averages of “level of agreement” of doctoral fellow’s program satisfaction. Overall, doctoral fellows agree that they are satisfied (*median*) with their progress towards doctoral milestones and their two doctoral degree programs, with slight dips in 2021; when looking at mean responses, most of these students indicate either “agree” or “somewhat agree” that they’re satisfied with academic progress and their doctoral degree programs. Interestingly, doctoral fellows level of agreement on their satisfaction with program activities, specifically mentoring and community shift over time. Fellows’ level of agreement that they are satisfied with the NRT community dropped from “strongly agree” to “somewhat agree” over the period, while fellows’ level of agreement that they are satisfied with their faculty mentorship increased from “somewhat agree” to “agree” over the same period.

Next, doctoral fellows were asked to indicate their level of agreement on whether the CNS NRT program had positively impacted their academic research skills during a given year. The research skills are assessed across five factors: writing, presentation, technical skills (e.g., improve computational and statistical modeling and methods), grant writing, and networking. Doctoral fellows’ interdisciplinary orientations were assessed using transdisciplinary orientation (TDO) scale [39], which was initially developed for characterizing scientific publications and transdisciplinary. TDO characterizes intrapersonal skills, behaviors, values, attitudes and beliefs that emerge over a scientist’s career that influence their participation in interdisciplinary research projects that tackle complex problems emerging in science [40]. The TDO scale is broken into two sets of factor variables that align with 1) transdisciplinary conceptual skills and behaviors and 2) transdisciplinary values, attitudes and beliefs.

**Fig 7:**
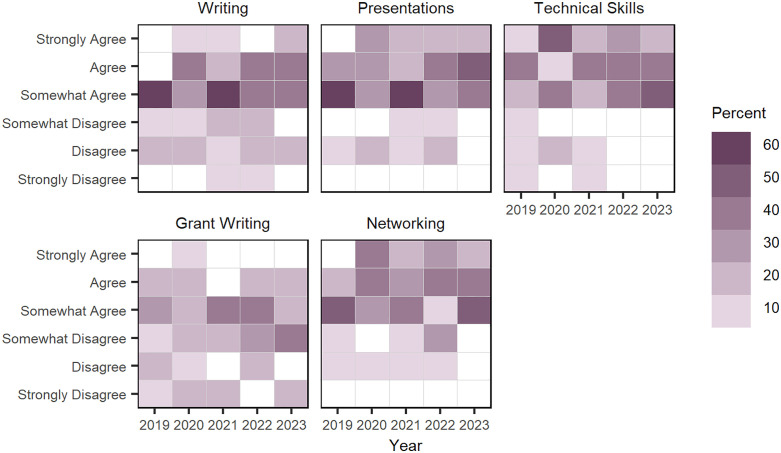
Doctoral fellows’ “level of agreement” that CNS NRT program has a positive impact on their research skills, 2019–2023. Each square is shaded to indicate the percentage of doctoral fellows who agree that the program has positively influenced their research skills in five areas.

Overall, doctoral fellows had mixed levels of agreement on the positive impact of the CNS program across research skills. For instance, during the survey period, most students only “somewhat agree” that the program improved their writing skills; while they tended to “agree” on the program’s impact on their presentation, technical skills and networking. Qualitative responses include: “I have had a great experience with the NRT. I could not do the research I’m interested in without the structure that allows me to be in two departments, as well as the support that allows me to take this many classes.”; “When you tackle problems within your own subfield you imagine people in other fields have not addressed those issues -- often they have, and there are reasons why no one has done what you thought was easy to do. On the other hand, I feel like I am better informed and closer to making those connections than I was one year ago, and am positive that they are feasible, albeit not necessarily easy.”; and “My biggest issue with the program is a lack of training in the actual methods and tools of complex systems.” The NRT leadership responded to the last comment by encouraging affiliated faculty to train in advanced methods and tools as not all of them can be covered in regular courses. Last, students had the least agreement around the program’s impact on their grant writing skills. Doctoral fellows’ overall “level of agreement” is visualized as Likert-heatmaps in Fig 7; see Table B in S2 File annual average “level of agreement” for research skill items.

**Fig 8:**
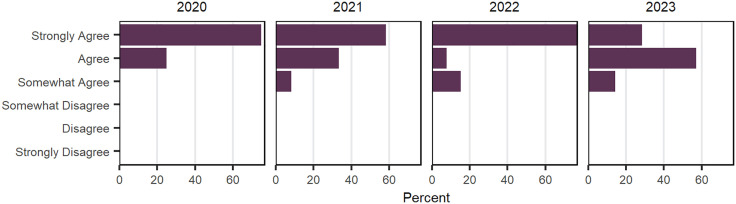
Doctoral fellows’ “level of agreement” on the CNS NRT program’s impact on interdisciplinary orientation, 2019–2023. Each bar represents the percentage of doctoral fellows who share a level of agreement that the CNS NRT program has positively impacted their interdisciplinary skills and values.

**Fig 9:**
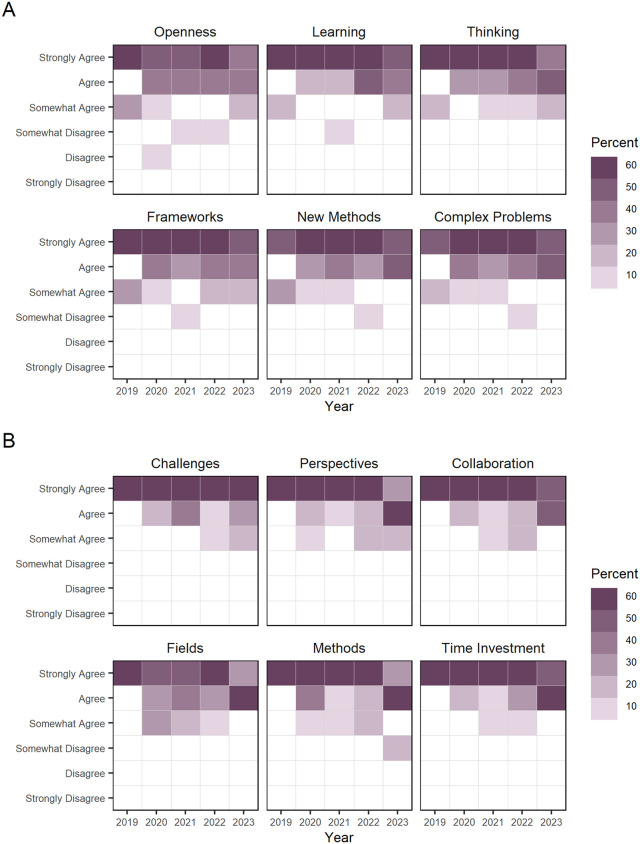
Doctoral fellows’ “level of agreement” on the CNS NRT program’s impact on interdisciplinary orientations, 2019–2023. Each square is shaded to show the percentage of doctoral fellows’ who share a level of agreement of the CNS NRT program’s impact on A) six interdisciplinary values, attitudes and beliefs factors and (B) six interdisciplinary conceptual skills and behaviors factors. Data is provided in S1 Dataset, S1B Table.

While there was overwhelming agreement that the program positively impacted students’ interdisciplinarity orientations; however, there was slight variation in how students responded to different conceptual skills and behaviors and factors related to interdisciplinary values, attitudes and beliefs (see Fig 8). Doctoral fellows’ self-assessment of program’s impact on factors associated with their interdisciplinary orientation are visualized in Fig 9. The interdisciplinary value of “Openness” showed multiple years where students showed high variance in the responses that the program had positively impacted this factor. Likewise, the interdisciplinary conceptual skill factor “Fields” tended to have more variation in the intensity of agreement than other skills or values between 2020 and 2022. Last, participants’ “level of agreement” dropped from “strongly agree” to “agree” for most factors in 2023.

#### CNS NRT mentorship activities.

Mentorship is a key component of the CNS NRT program, where doctoral fellows are paired with two faculty members, the first coming from the Complex Network and Systems doctoral program, and the second coming from the second doctoral program the student has identified. Faculty mentors serve an advisory role for doctoral students in the program, provide guidance related to course selection, acculturation to scientific research practices, provide doctoral research experiences, offer recommendations for development of technical skills, and introduction to grant writing and support to students pursuing funding opportunities.

The survey was designed to ask both faculty mentors and doctoral fellows about the impact that the mentorship program had on students’ research skills. Doctoral fellows were also asked to evaluate the quality of their mentors across a set of behaviors associated with successful mentoring relationships [41]. Both groups were invited to provide additional comments on their mentoring experiences. Qualitative responses include: “It really has been my mentors, plus the affordance to take risks, along with colloquium that has allowed this [to make an impact].”; “Interdisciplinary programs are difficult, but it is really helpful for grad students as it gives them access to tools that they would have otherwise not have (singular mentorship in other departments means that you only learn the techniques of your mentor).”; and “I think learning CNS methods are particularly useful, and other kinds of “general skills training” (grant writing, mentorship, publishing) can come from the department level and not from the NRT program.”. Regarding the last comment, the Luddy School now offers several grant writing, publication, and scholarly communication workshops; mentorship needs to come from thesis advisors and others in the department(s) that will confer the degree.

**Fig 10:**
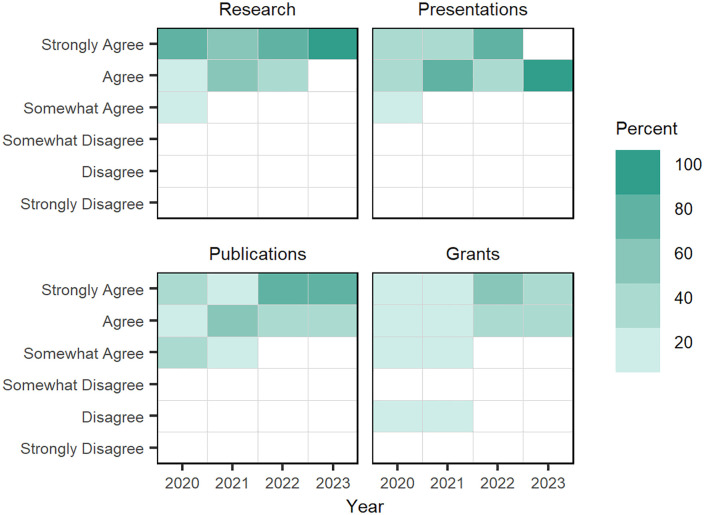
Faculty agreement that mentorship has a positive impact on doctoral students’ research skills, 2019–2023. Each square is shaded to represent the percentage of faculty members’ level of agreement that their mentorship has positively impacted their doctoral fellows’ research skills in four areas.

**Faculty assessment of mentorship impact on doctoral fellow research skills:** From 2020 to 2023, NRT faculty mentors were asked about the impact of their mentoring activities on doctoral fellows’ research skills. Faculty mentors were asked to indicate their agreement that mentoring positively impacted doctoral students’ research, presentation, publication, and grant writing skills. Overall, faculty mentors tended to “agree” or “strongly agree” that their mentorship activities had positive impacts on their doctoral students’ skills (see Table C in S2 File for faculty’s item response counts and average Likert responses scores). When faculty “agreement levels” are visualized across these skill areas (see Fig 10) between 2020 and 2021, grant writing emerges as a skill set where faculty members show greater variation in their agreement ratings.

**Doctoral assessment of mentorship impact on research skills:** From 2021 to 2023, doctoral fellows were asked about the impact faculty mentoring activities had on their research skills. Like faculty mentors, doctoral students were asked to indicate their agreement that mentoring positively impacted their research, presentation, publication, and grant writing skill development; however, the survey also asked students about mentorship’s impact on their collaboration and interdisciplinary skills.

**Fig 11:**
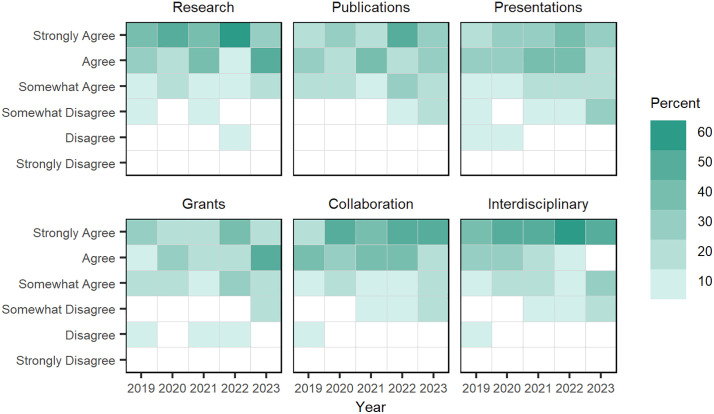
Doctoral fellows’ “level of agreement” that mentorship has a positive impact on doctoral fellows’ research skills, 2019–2023. Each square is shaded to represent the percentage of doctoral fellows’ level of agreement that their mentorship has positively impacted their doctoral fellows’ research skills in six areas.

Overall, doctoral fellows tended to “strongly agree” that their mentorship activities had positive impacts on their research, interdisciplinary, and collaboration skills, while having weaker levels of agreement on presentation, publication, and grant writing skills; item response counts and mean Likert ratings are found in Table D in S2 File). Doctoral fellow “agreement levels” are visualized across these six skill areas (Fig 11) and show that students consistently had strong agreement on the impact mentors had on student’s research, interdisciplinary, and collaboration skills.

**Doctoral assessment of mentorship quality:** Each year, doctoral fellows were asked to assess the quality of their mentors based on their interactions from the previous year. Mentorship quality was evaluated on eleven areas, which include: accessibility, acknowledges hard work, approachability, challenging, constructive feedback, has domain expertise, offers guidance, has integrity, provides resources, is responsive to students, and is supportive of students (13).

**Fig 12:**
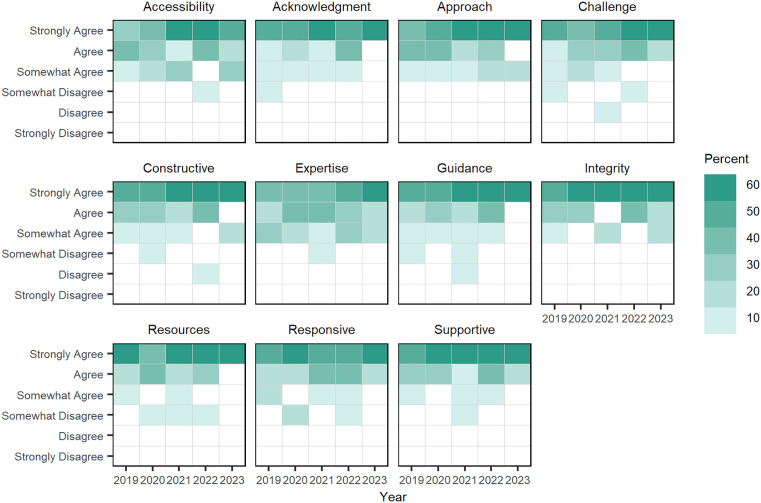
Doctoral fellows’ “level of agreement” on mentorship quality, 2019–2023. Each square is shaded to represent the percentage of doctoral fellows’ who share a level of agreement on the quality of faculty mentorship across 11 factors.

Doctoral fellows indicated their level of agreement with statements about each of these qualities. Overall, doctoral fellows strongly agreed that their faculty mentors demonstrated these qualities in their relationships (see Table E in S2 File). Doctoral fellow “agreement levels” on the quality of mentorships are visualized for 2019–2023 in Fig 12, showing students’ level of agreement on the quality of their mentors improved in later years of the survey. However, this trend may be caused by lower participation rates by students in later years.

Qualitative survey responses discuss difficulties that arise when faculty from different departments advise one student; highlight the impact of COVID-19 on the frequency and quality of meetings with mentors, fellow students, and others; and they document the fact that there are different PhD stages (e.g., identification of research topic and best mentor(s) while taking classes; review of existing work; thesis proposal; and thesis work and defense) that benefit from different types of guidance and mentorship.

#### Impact of COVID-19 pandemic.

**Fig 13:**
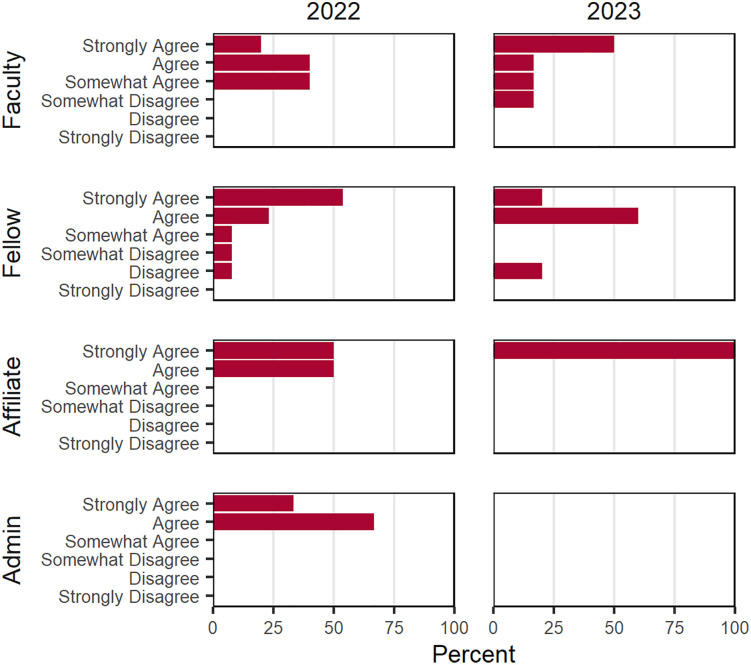
CNS NRT members’ reflection on the impact of Covid-19 the CNS NRT program, 2022−2023. Each bar represents the percentage of doctoral fellows who share a level of agreement that the CNS NRT program was impacted by the COVID-19 pandemic. Data is provided in S1 Dataset, S1C Table.

As has been documented across the news and scholarly literature, the COVID-19 pandemic that began in 2020 had a significant impact on the CNS NRT program and its members. In 2022 and 2023, the survey asked participants to assess the impact of the pandemic on the program activities. Responses from the CNS NRT member groups for each year are visualized using bar graphs in Fig 13. Overall, members agreed that the pandemic impacted the NRT program. Participants were asked to provide comments to help clarify their responses; qualitative responses from 2021 include: “Given how the NRT was virtual this year, it was difficult to meet with students organically outside of the colloquiums and lab meetings. I appreciate the effort taken by the staff and faculty to accommodate us during the pandemic.”; and, during 2021: “Because I joined during the pandemic, I feel like many of my options were very limited, including interacting with other NRT students, talking with visiting speakers, getting to know NRT-affiliated faculty, and exploring my research options. Regardless of how much enthusiasm I or the speakers bring to a Zoom meeting, I never get as much out of them as in-person events.”; and “Meeting on Zoom is just not the same as meeting in person. You won’t have any of the unscripted, one-on-one conversations with faculty that you happen to run into at and around the event”.

### Publication analysis

In addition to the annual survey, doctoral fellows and summer research affiliate students are required to report their research activities from the prior year, including citations for research where they earned authorship credit. Research publications provide documentary evidence of graduate students’ research skills development, including technical skills, writing and presentation skills, and positive mentorship outcomes that provide opportunities for collaboration and development of interdisciplinary values relevant to complex networks and systems science.

**Fig 14:**
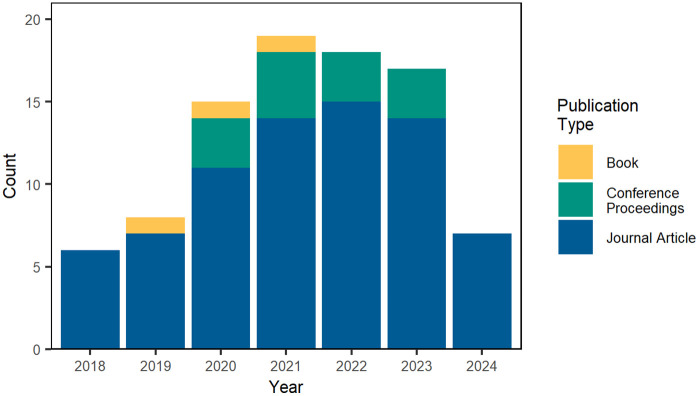
Annual CNS NRT publications, by type. This stacked bar graph shows the number of research articles published by NRT members between 2018 and 2024. Each bar segment represents a different type of publication, including book chapters, conference papers, and journal articles.

Between 2019 and 2023, NRT members reported 63 publications that document research efforts funded, in part, by the CNS NRT. In August 2024, a secondary search of the Web of Science (WoS) and Google Scholar database using CNS NRT NSF grant award number (1735095) found an additional 49 publications, including 7 articles published in 2024. In total 112 publication citations linked to the CNS NRT were identified. Examination of the document types and titles identified 22 pre-print articles that duplicated a later publication within the data set; and these 22 pre-prints were excluded from analysis. In total 90 articles were included in the analysis; a bar graph shows the annual publication counts, by publication type in Fig 14.

#### Member group participation.

Analysis of the publication author names led to the identification of 239 unique authors, of which 49 authors (20.5%) are members of the CNS NRT community; group level statistics, which includes publication counts, author counts, and publication rates, are presented in Table 7. Interestingly, 60% of faculty published research articles with NRT doctoral fellows or affiliate students, while 51.4% of doctoral fellows and 36.8% of summer research affiliate students demonstrated research skills through these publications. The remaining 190 authors (79.5%) are collaborators who worked on 72 of the 90 publications associated with the NRT.

**Table 7:** CNS NRT member group authorship statistics.

NRT Role	Membership	Authors	Percent of Group with Publication	Publications
Faculty	40	24	60.0%	65
Doctoral Fellow	35	18	51.4%	56
Affiliate Student	19	7	36.8%	22
Collaborator	NA	190	NA	72

**Fig 15:**
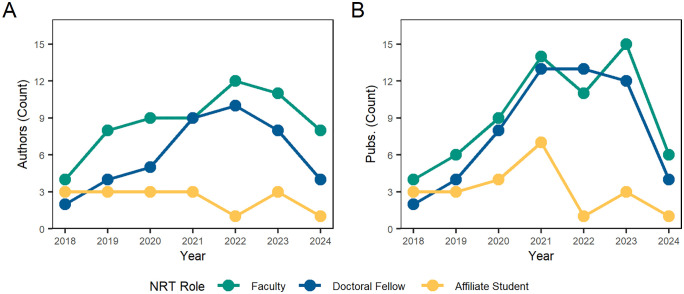
Annual count of authors and publications, by CNS NRT role. Inset A presents the unique number of authors that published research during a given year by NRT role. Inset B presents the number of research papers published each year by NRT role.

A temporal analysis of author roles between 2018 and 2024 is shown in Fig 15. NRT faculty and doctoral fellow authorship participation grew annually until peaking in 2022. Similarly, NRT faculty and doctoral fellow publication counts grew quickly in the first two years of the program, and then remained high between 2021 and 2023, with a significant drop off in 2024 as funding for the program ended. Summer research affiliate students saw their highest number of publications (7) in 2021. Summer affiliate student publication lowered in the final two years of the program.

#### Publication citations and journal subject categorization.

Of the 90 publications, 64 research articles and conference papers were indexed in WoS, which provides additional information about publication data, including publication citation analysis, subject categories used to classify journals, and author’s organization affiliations. The results of the Web of Science citation, subject categorization, and author affiliation analyses are available in S2 Dataset.

The 64 publications indexed in WoS were cited a total of 1,921 times between 2018 and 2024, with papers receiving an average of 30 citations, and an *h*-index of 20. These citations came from 1,746 citing articles, excluding self-citations. The highest cited paper is “The spread of low-credibility content by social bots,” published by *Nature Communications* in 2018, includes two NRT faculty authors; the paper has been cited 522 times (27% of citations). (https://doi.org/10.1038/s41467-018-06930-7). Four other papers have over 90 citations.

All publications indexed across multiple collections representing broad areas of science and levels of research are combined into the WoS Core Collection. CNS NRT papers are indexed primarily across 6 collections: 47 articles indexed in the Science Citation Index Expanded, 19 articles indexed in the social sciences citation index, 4 articles are indexed in the Emerging Sources Index, and the remaining 6 articles are indexed in book and conference proceedings indices. In addition to indexing, WoS assigns each publication at least one of 254 subject categories based on the disciplinary and subject area associated with a journal title; journals and publications in this data set are assigned between 1 and 5 WoS subject categories. CNS NRT papers fall within 44 subject categories. The top subject category, “Multidisciplinary Sciences,” has 23 papers associated with it; other top subject categories are found in Table 8. A review of the categories shows that 7 of the 44 subjects categories include either multi- or interdisciplinary in their label (e.g., “Physics Multidisciplinary”, “Social Sciences Interdisciplinary”, and “Agriculture Multidisciplinary”); these categories were assigned 38 times across publications indexed in WoS. A review of the scope notes for the WoS subject categories shows that the terms multi- and interdisciplinary are used interchangeably; for example, “Physics, Multidisciplinary” is applied to any journal title that “...covers resources having a general or interdisciplinary approach to physics.” The broadest category, “Multidisciplinary Sciences” is reserved for journals with a broad coverage of major scientific disciplines and includes journals like *Nature* and *Science*.

**Table 8:** CNS NRT publication Web of Science subject categorization.

Web of Science Categories	Publications	Percent
Multidisciplinary Sciences	23	35.9%
Neurosciences	6	9.4%
Mathematical Computational Biology	5	7.8%
Physics, Multidisciplinary	5	7.8%
Biochemical Research Methods	4	6.3%
Communication	4	6.3%
Computer Science, Interdisciplinary Applications	4	6.3%
Neuroimaging	3	4.7%
Radiology Nuclear Medicine Medical Imaging	3	4.7%
Computer Science Information Systems	2	3.1%
Geography	2	3.1%
Health Care Sciences Services	2	3.1%
Mathematics, Interdisciplinary Applications	2	3.1%
Medical Informatics	2	3.1%
Psychology Clinical	2	3.1%
Regional Urban Planning	2	3.1%
Social Sciences, Interdisciplinary	2	3.1%

#### Co-authorship network analysis.

Next, we applied co-authorship network analysis methods to identify key authors within the network and understand author collaboration and communities that emerged over the course of the NRT program and research efforts. Two networks were created to represent two time periods for the NRT: the first network represents co-authorship of 48 publications from the period between 2018 and 2021, covering the first half of the program; and the second co-authorship network represents 90 articles published between 2018 and 2024. Both networks are made of undirected and weighted relationships between authors, where weight equals the number of publications that they both authored. Authors’ names are withheld from the network visualizations labels to provide a degree of anonymity; however, the CNS NRT publications data used to create these networks includes author names. The authors in the network data and visualizations are identified using a composite name that includes their NRT group membership and number. Both networks label the same set of authors, which includes 19 authors with at least four publications in the data.

**Fig 16:**
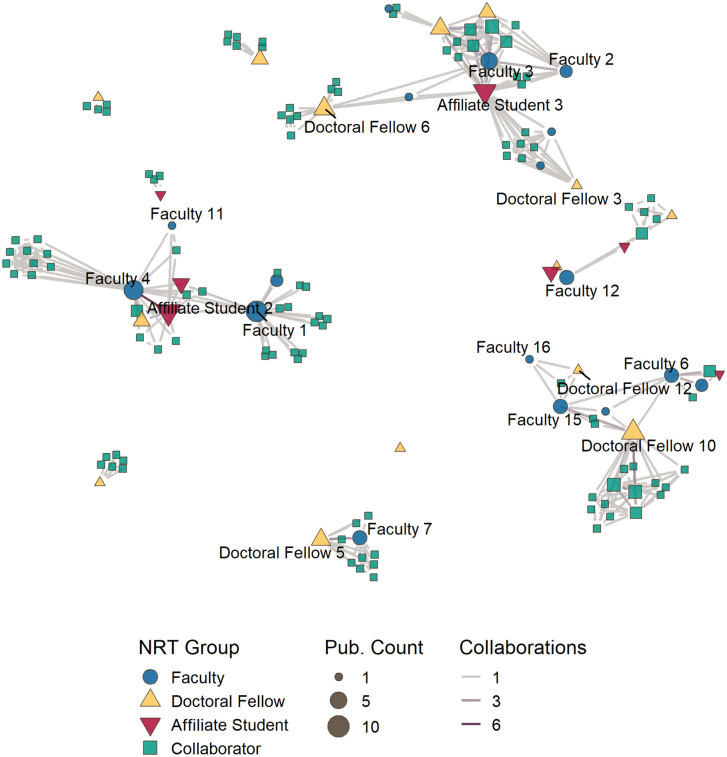
CNS NRT co-authorship network for 2018–2021. 149 authors and their 466 relationships associated with 48 research articles published during this period are represented in this network. Author nodes color and shape shows the NRT group membership, while node size indicates the number publications published during this period. Authors with at least 4 publications between 2018–2024 are highlighted in this network.

**Fig 17:**
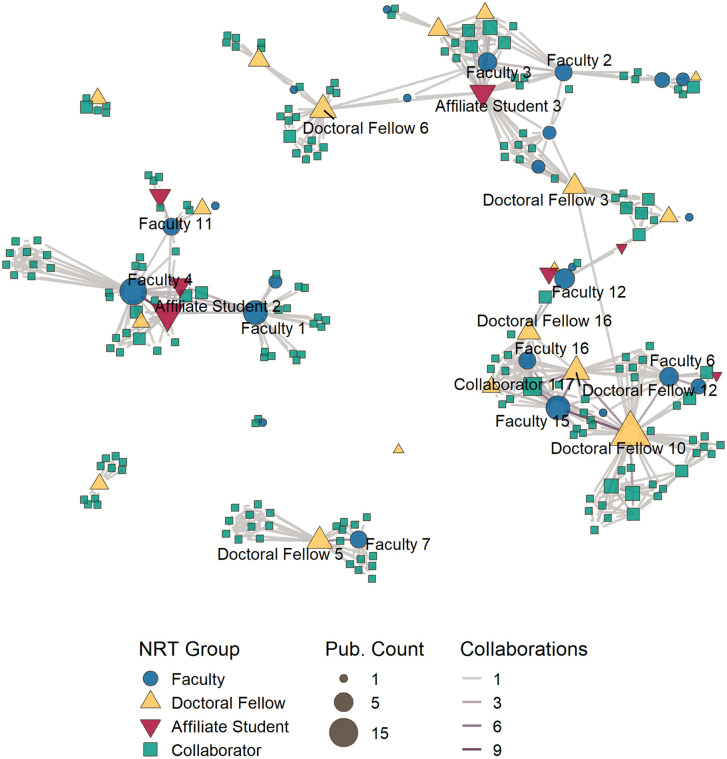
CNS NRT co-authorship network for 2018–2024. 239 authors and their 806 relationships associated with 90 research articles published during this period are represented in this network. Author nodes color and shape show the NRT group membership, while node size indicates the number of publications published during this period. Authors with at least 4 publications between 2018–2024 are highlighted in this network.

Between 2018 and 2021, the CNS NRT co-authorship network included 149 authors with 466 relationships, which included 17 NRT faculty members, 14 doctoral fellows, 7 summer research affiliates, and 111 collaborators (Fig 16). From 2022 to 2024, the co-authorship network grew to a total of 239 authors and 806 relationships (Fig 17). Over the three-year period, the network added 79 collaborators, seven NRT faculty and four doctoral fellows; no new summer research affiliate students joined the network. The initial network represents 62.3% of the total authors and 57.8% of the relationships established by the final network. The density of the co-authorship network dropped from 0.042 to 0.283 between the 2021 and 2024 networks.

The co-authorship network coalesced from ten to seven components in the years between 2021 and 2024. The largest component from the 2018–2024 network is composed of four components present in the 2021 instance of the network, and includes 136 authors (56.9%), including 17 faculty members, 12 doctoral fellows, 4 affiliate students, and 103 collaborators. The network includes one clique that did not include any NRT doctoral fellow or summer research affiliate as authors. When the 2024 network is restricted to authors with at least two publications, the network reduces to 59 authors and 156 relationships. NRT members make up a greater proportion of authors in these networks; collaborators membership dropped from 79.5% of authors to 64.4% in the filtered network, while NRT author membership grew from 20.5% to 35.6% of the network.

Analysis of the NRT members in the network shows that 16 of 24 NRT faculty members (64%); 15 of 18 doctoral fellows (83.3%); and 5 of 7 summer research affiliate students (71.4%) have at least two publications. The top contributing author is a doctoral fellow, who wrote 17 papers. Examination of the author relations (Table 9) show that between 2021 and 2024 collaborators were involved with between 89.5 and 60.8% of all relationships in the NRT co-authorship network, while faculty were involved with between 27% and 17.4% of relationships and doctoral fellows were involved in 21.9% and 17% of relationships during the same period. Doctoral fellows showed the highest rate of growth in their network relationships during this period, growing from 102 to 205 co-author relationships.

**Table 9:** CNS NRT relationship statistics, by author role.

NRT Group	2018–2021		2018–2024		Growth Rate
	Relationships	Percent	Relationships	Percent	
Faculty	126	27.0%	210	17.4%	66.7%
Doctoral Fellow	102	21.9%	205	17.0%	101.0%
Affiliate Student	51	10.9%	58	4.8%	13.7%
Collaborators	417	89.5%	732	60.8%	75.5%

Next, we analyzed relationships by the authors’ NRT group membership pairings, which are found in Table 10**.** Nearly half of all collaborations were between non-NRT affiliated authors, with the next three highest percentage of pairings involving NRT members and collaborators (i.e., faculty and collaborators, doctoral fellows and collaborators, and affiliate student researchers and collaborators). Further examination of the results shows that NRT faculty and doctoral fellow collaborations makeup 3.4% and 3.7% of all co-authorship relationship pairs in 2021 and 2024, with there being a total of 30 faculty and doctoral student relationships established by 2024. NRT faculty and summer research affiliate students’ collaborations dropped from 3.0% to 1.9% of co-authorship pairs from 2021 to 2024, with a total of 15 relationships established by 2024.

**Table 10:** CNS NRT member group relationship-pair statistics.

NRT Member Group Relationship Pair	2018–2021		2018–2024	
	Count	Percent	Count	Percent
Faculty – Faculty	11	2.4%	15	1.9%
Faculty – Doctoral Fellow	16	3.4%	30	3.7%
Faculty – Affiliate Student	14	3.0%	15	1.9%
Faculty – Collaborator	85	18.2%	150	18.6%
Doctoral Fellow – Doctoral Fellow	1	0.2%	7	0.9%
Doctoral Fellow – Affiliate Student	6	1.3%	6	0.7%
Doctoral Fellow – Collaborator	79	17.0%	162	20.1%
Affiliate Student – Affiliate Student	1	0.2%	1	0.1%
Affiliate Student – Collaborator	30	6.4%	36	4.5%
Collaborator – Collaborator	223	47.9%	384	47.6%

#### Co-author affiliations.

An analysis of author affiliations identified by WoS shows that the CNS NRT collaborators are associated with 120 different organizations; note that 13 affiliations were excluded from the results that duplicated an organization at the system level, and 1 excluded organization included duplicated Indiana University Bloomington at the school level. CNS NRT collaborators come from 54 US organizations and 66 international organizations. Most collaborators are affiliated with 87 different universities and colleges. However, 33 of the organization affiliations include research labs, foundations, banks and hospitals. The top five CNS NRT collaborator affiliations are found in Table 11.

**Table 11:** Top collaborator organizations identified by WoS.

Organizations	Publications
Instituto Gulbenkian de Ciencia	11
Binghamton University Suny	7
Coordenacao de Aperfeicoamento de Pessoal de Nivel Superior (Capes)	5
University of Cambridge	5
Northeastern University	3
Ohio State University	3
University of Washington Seattle	3

## Discussion

The primary purpose of the evaluation is to assess how well the CNS NRT program achieved its short-term goals in training a diverse cohort of graduate students in dual doctoral degree programs in both complex networks and systems and a secondary related degree. Doctoral fellows were supported in the process by strong mentoring relationships that sought to incorporate research experiences that developed the students’ research skills and interdisciplinary culture. Secondarily, the program hoped to increase awareness of the application of complex networks and systems methods to a wide variety of natural and social science disciplines. Overall, when surveyed, CNS NRT members agreed that the program met its educational goals and disseminated its research activities well. However, participants showed disagreement on whether the program improved local and national awareness of the NRT program and, more broadly, for complex networks and science as a discipline. Faculty members tended to agree that the program had increased awareness of complex networks and systems and the CNS program at IU; doctoral students tended to not agree that these goals were met.

In terms of the educational goals, the CNS NRT program has successfully graduated seven (20%) doctoral students, with a remaining 15 doctoral fellows on track to complete their degrees by 2028. While nine students left the program, all of these students either remained enrolled in a doctoral or master’s program at Indiana University or transferred to a doctoral program at another university to complete their degrees. The NRT succeeded in creating large cohorts of fellows working in dual degrees in both the cognitive science and informatics doctoral programs. The gender make-up of doctoral fellows admitted and enrolled to the program (60:40 male to female) demonstrates progress towards gender parity in CNS program enrollments within the IU informatics doctoral program, which between 2012 and 2016 had an overall enrollment ratio of 66:36 male to female students. However, the limited racial and ethnic diversity of doctoral fellows show where the CNS program and informatics program needs to improve its recruitment and admissions process.

CNS NRT doctoral fellows are satisfied with the program in terms of their progress through their doctoral degree and with the CNS and their secondary doctoral degree programs. While students’ agreement varies on the impact of the CNS NRT program on their academic research skills, doctoral fellows overwhelmingly agree that the program has had a positive impact on their interdisciplinary skills and personal values. Additionally, doctoral fellows agreed that their mentors were of high quality and that their mentors helped improve their skills in research methods, collaboration, and interdisciplinarity. Doctoral students had lower levels of agreement on the NRT program and mentors’ impacts on grant writing, presentation and publications skills.

A limiting factor in interpretation of the survey data are the low participation rates by NRT faculty members between 2019 and 2023. Doctoral fellows had the highest number of participants each year, as 1) doctoral students are required to participate by their funding contracts, and/or 2) they are personally invested in positive outcomes from the NRT program. This latter motivation for participation also likely explains the doctoral fellows’ higher variance in their agreement across questions and items than in faculty, affiliate student researchers and administrators.

Publication data provides evidence of the NRT members’ efforts to publicize CNS research to increase local, national, and international awareness of complex networks and systems research, as well as providing evidence of the program’s success in supporting doctoral fellows’ research skills, specifically, in writing, presentation, technical skills and collaboration. Analysis results show NRT faculty and students engaged in highly collaborative, interdisciplinarity research over seven years of data analyzed. On average, the CNS NRT program published 12–13 articles each year across a wide range of subject areas and disciplines and collaborated with 190 scholars from 120 different organizations across the world.

Over half of doctoral fellows published research in an academic journal or conference proceeding during their time in the program while 46% of doctoral fellows had at least two publications. While this is a positive outcome, a large portion of the NRT fellows (48%) either failed to report publications to the evaluation committee or have yet to publish research work. Indeed, poor reporting of research outcomes by NRT doctoral fellows and summer research affiliates included 27 missed publications that were later found in a secondary search of the NRT program’s grant identifier and included in the publication analysis. It is important to consider that doctoral student participation in the scholarly communications process may be impacted by several factors, including the working relationship the student has with their academic mentors, differences in the normative practices of the academic disciplines they are studying, or personal issues. Plus, the COVID-19 pandemic negatively impacted interactions between students and faculty.

The challenge of obtaining complete assessments from participants (e.g., low participation rates for surveys, incomplete reporting of research outcomes) is not unique to the CNS NRT program. However, the interdisciplinary, long-term nature of the CNS NRT program added novel challenges. Student record keeping is typically performed at the department level (here, 10 + departments) by staff that changes multiple times over the duration of the program; records are kept in spreadsheets and online systems in diverse formats that are incompatible. While information on course and degree completion is easier to access, information on publications, changes in mentorship, participation in events, etc. is hard to track across departments.

The CNS NRT evaluation committee navigated a variety of challenges including changes in the evaluation management team, which led to adapting work outlined in the initial evaluation plan and reporting. Turnover in administrative staffing in the informatics program and IT offices led to issues accessing data from the student information system, which impacted the quality and timeliness of doctoral students’ progress through academic milestones and advising data. The COVID-19 pandemic led to overnight changes in staffing and teaching methods in the spring of 2020 that led NRT faculty, students, and administrators to make significant adaptations to their personal and work lives, which highlights the resilience of program participants.

Another significant limiting factor in assessing doctoral degree programs is the individualized nature of graduate education that has a limited pool of students, who do not have easily comparable academic research and training experiences. The assessment of the CNS NRT does not include two analyses that were made during the annual evaluations, including an analysis of doctoral fellows’ progress towards their academic milestones and an analysis of the mentor advising networks. Both analyses rely on student level data that captures details about academic performance and relationships that have data privacy protections. Due to the limited size of participants and annual cohort pools within the CNS NRT and similar doctoral programs, the risk of re-identification of individuals’ identities and confidential academic records is too high, and the results are not included here.

Going forward, CNS NRT leadership and the evaluation committee are interested to assess the program’s success and impact towards its mid- and long-term outcomes, which includes doctoral fellows’ job placement in academic and industry settings, their post-doctoral research activities and impact, and future doctoral students they train. With a growing number of graduates, the CNS NRT program could use data from LinkedIn and IUs alumni database to study mid- and long-term impacts.

## Supporting information

S1 Table Complex networks and systems NSF research traineeship (CNS NRT) program outcomes. (XLSX)

S2 Table CNS NRT core faculty affiliations. This table shows the number of faculty affiliations by department at IU.
(CSV)

S1 File CNS NRT annual survey documentation. This zip file contains documentation for the annual survey that includes table (Table A) that lists questions with annual indices that indicate if a question was asked, and a document (Text A) that provides definitions for the Likert Scales used during the annual survey.
(ZIP)

S2 File CNS NRT annual survey Likert response descriptive statistics for select question sets. Table A. CNS NRT doctoral fellows’ average “level of agreement” that they are satisfied with the program and their progress towards their doctoral degree (2019–2023). Table B. CNS NRT Doctoral Fellows’ average “level of agreement” that the CNS NRT program has positively impacted their research skills (2019–2023). Table C. Faculty members’ average “level of agreement” that mentorship has positively impacted doctoral fellows’ research skills (2020–2023). Table D. Doctoral Fellows’ average level of agreement that mentorship has positively impacted their research skills (2019–2023). Table E. Doctoral Fellows’ average level of agreement on mentorship qualities (2020–2023).
(DOCX)

S1 Dataset CNS NRT annual survey analysis, supporting data for Figs 6, 9 and 13. Table A. CNS NRT member assessment of program goals. Calculations of mean Likert responses values and SD for respondents’ assessment that the CNS NRT has met its program goals during a given year. Table B. CNS NRT doctoral fellow interdisciplinary orientation. Calculations of mean Likert responses values and SD for factors in the transdisciplinary orientation scales. Table C. CNS NRT Covid Impact. Calculations of mean Likert responses values and SD of respondents’ assessment of COVID-19 pandemic on CNS NRT program activities.
(ZIP)

S2 Dataset CNS NRT Web of Science publication analysis. This zip file contains four data tables collected from Web of Science (WOS) that were used in part or support results presented in the publication analysis section of this paper. The dataset includes the following: Table A. CNS NRT publication identifiers (2018–2024). Table B. CNS NRT publications WOS author affiliations. Table C. CNS NRT publications WOS citation report (2018–2024). Table D. CNS NRT publications WOS journal subject categories. Table E. WOS subject category scope notes.
(ZIP)
