# Supplementary material for: Preparing the next generation of Complex Networks and Systems scientists: Evaluation results for the Complex Networks and Systems NSF research training program at Indiana University
Source: PLoS One. 2026 Jan 27;21(1):e0334779. doi: 10.1371/journal.pone.0334779 (PMC12843521; doi:10.1371/journal.pone.0334779)
Supplement: S1 File — This zip file contains documentation for the annual survey that includes table (Table A) that lists questions with annual indices that indicate if a question was asked, and a document (Text A) that provides definitions for the Likert Scales used during the annual survey. (ZIP) [file pone.0334779.s003.zip › TextA.docx]

**Appendix A**

This document collects tables that provide additional analysis of CNS NRT annual survey for 2019-2023.

Unless otherwise indicated, participants were asked to indicate their level of agreement using a six-level Likert scale that includes the following values: Strongly Agree (6), Agree (5), Somewhat Agree (4), Somewhat Disagree (3), Disagree (2), Strongly Disagreed (1); participants may also select Not Applicable (0) if they do not have a response, which are excluded from the analysis. Descriptive statistics are calculated after by converting Likert values to a numeric score (i.e., the values in the parenthesis above).

## Survey Metadata

**Table 1.** Count of questions asked of participants during the annual survey, by NRT membership group, for each year between 2019 and 2023.

| **Group** | **2019** | **2020** | **2021** | **2022** | **2023** |
| --- | --- | --- | --- | --- | --- |
| All | 4 | 5 | 7 | 33 | 33 |
| Doctoral Fellow | 57 | 59 | 59 | 59 | 59 |
| Faculty | 7 | 8 | 8 | 8 | 8 |
| Affiliate | 0 | 8 | 8 | 8 | 8 |
| **Total Questions** | **68** | **80** | **82** | **108** | **108** |

## Overall Program Goals and Help with Dissemination and Awareness

**Table 2.** CNS NRT membership groups’ average “level of agreement” that the program has achieved its goals and helped disseminate research in and increase local and national awareness of complex network and systems, for the period 2019-2023.

| **Item** | **Role** | **Responses** | **Median** | **Mean** | **SD** |
| --- | --- | --- | --- | --- | --- |
| Overall Goals | Faculty | 48 | 5.5 | 5.4 | 0.89 |
|  | Fellow | 65 | 5 | 4.8 | 0.88 |
|  | Affiliate | 12 | 6 | 5.8 | 0.45 |
|  | Admin | 19 | 5 | 5.2 | 1.17 |
| Disseminate Research | Faculty | 54 | 5 | 5.2 | 0.97 |
|  | Fellow | 63 | 5 | 4.5 | 1.13 |
|  | Affiliate | 13 | 6 | 5.6 | 0.65 |
|  | Admin | 15 | 5 | 5.4 | 0.51 |
| Local Awareness | Faculty | 50 | 6 | 5.4 | 0.96 |
|  | Fellow | 63 | 5 | 4.7 | 1.26 |
|  | Affiliate | 13 | 6 | 5.6 | 0.51 |
|  | Admin | 19 | 5 | 5.1 | 1.13 |
| National Awareness | Faculty | 48 | 5 | 4.9 | 1.12 |
|  | Fellow | 58 | 4 | 3.8 | 1.39 |
|  | Affiliate | 9 | 4 | 4.6 | 0.73 |
|  | Admin | 15 | 5 | 4.1 | 1.46 |

## Mentorship

**Table 3.** Faculty members' average "level of agreement" that mentorship has positively impacted doctoral fellows' research skills, for 2020-2023.

| **Item** | **Year** | **Responses** | **Median** | **Mean** | **SD** |
| --- | --- | --- | --- | --- | --- |
| Grants | 2020 | 5 | 5 | 4.6 | 1.673 |
|  | 2021 | 4 | 4.5 | 4.2 | 1.708 |
|  | 2022 | 3 | 6 | 5.7 | 0.577 |
|  | 2023 | 2 | 5.5 | 5.5 | 0.707 |
| Presentations | 2020 | 9 | 5 | 5.3 | 0.707 |
|  | 2021 | 6 | 5 | 5.3 | 0.516 |
|  | 2022 | 4 | 6 | 5.8 | 0.5 |
|  | 2023 | 3 | 5 | 5 | 0 |
| Publications | 2020 | 8 | 5 | 5 | 0.926 |
|  | 2021 | 5 | 5 | 5 | 0.707 |
|  | 2022 | 4 | 6 | 5.8 | 0.5 |
|  | 2023 | 3 | 6 | 5.7 | 0.577 |
| Research | 2020 | 10 | 6 | 5.7 | 0.675 |
|  | 2021 | 6 | 5.5 | 5.5 | 0.548 |
|  | 2022 | 4 | 6 | 5.8 | 0.5 |
|  | 2023 | 3 | 6 | 6 | 0 |

**Table 4.** Doctoral fellows' average level of agreement that mentorship has positively impacted their research skills, for 2019-2023.

| **Item** | **Year** | **Responses** | **Median** | **Mean** | **SD** |  |
| --- | --- | --- | --- | --- | --- | --- |
| Research | 2019 | 9 | 5 | 5.1 | 1.054 |  |
|  | 2020 | 13 | 6 | 5.5 | 0.776 |  |
|  | 2021 | 13 | 5 | 5.2 | 0.899 |  |
|  | 2022 | 12 | 6 | 5.4 | 1.24 |  |
|  | 2023 | 6 | 5 | 5.2 | 0.753 |  |
| Publications | 2019 | 7 | 5 | 5 | 0.816 |  |
|  | 2020 | 10 | 5.5 | 5.3 | 0.823 |  |
|  | 2021 | 10 | 5 | 5.2 | 0.632 |  |
|  | 2022 | 12 | 5.5 | 5.1 | 1.084 |  |
|  | 2023 | 6 | 5 | 4.8 | 1.169 |  |
| Presentations | 2019 | 8 | 5 | 4.5 | 1.414 |  |
|  | 2020 | 12 | 5 | 5.1 | 1.165 |  |
|  | 2021 | 13 | 5 | 5 | 0.913 |  |
|  | 2022 | 12 | 5 | 5.1 | 0.996 |  |
|  | 2023 | 6 | 4.5 | 4.5 | 1.378 |  |
| Interdisciplinary | 2019 | 9 | 5 | 5 | 1.323 |  |
|  | 2020 | 15 | 6 | 5.5 | 0.743 |  |
|  | 2021 | 13 | 6 | 5.2 | 1.013 |  |
|  | 2022 | 12 | 6 | 5.5 | 1 |  |
|  | 2023 | 6 | 5 | 4.8 | 1.329 |  |
| Grants | 2019 | 7 | 5 | 4.7 | 1.496 |  |
|  | 2020 | 9 | 5 | 5 | 0.707 |  |
|  | 2021 | 6 | 5 | 4.7 | 1.506 |  |
|  | 2022 | 10 | 5 | 4.8 | 1.317 |  |
|  | 2023 | 6 | 5 | 4.7 | 1.033 |  |
| Collaboration | 2019 | 8 | 5 | 4.8 | 1.282 |  |
|  | 2020 | 15 | 6 | 5.5 | 0.743 |  |
|  | 2021 | 12 | 5 | 5.2 | 0.937 |  |
|  | 2022 | 12 | 5.5 | 5.2 | 0.965 |  |
|  | 2023 | 6 | 5.5 | 5 | 1.265 |  |

**Table 5.** Doctoral fellows' average level of agreement on mentorship qualities, for 2020-2023.

| **Item** | **Year** | **Reps.** | **Median** | **Mean** | **SD** |
| --- | --- | --- | --- | --- | --- |
| Accessibility | 2019 | 8 | 5 | 5.2 | 0.707 |
|  | 2020 | 14 | 5.5 | 5.4 | 0.745 |
|  | 2021 | 13 | 6 | 5.3 | 0.947 |
|  | 2022 | 12 | 6 | 5.4 | 0.9 |
|  | 2023 | 6 | 5.5 | 5.2 | 0.983 |
| Acknowledgment | 2019 | 8 | 6 | 5.2 | 1.165 |
|  | 2020 | 12 | 6 | 5.7 | 0.651 |
|  | 2021 | 10 | 6 | 5.7 | 0.675 |
|  | 2022 | 11 | 6 | 5.5 | 0.688 |
|  | 2023 | 4 | 6 | 6 | 0 |
| Approach | 2019 | 9 | 5 | 5.3 | 0.707 |
|  | 2020 | 15 | 6 | 5.5 | 0.64 |
|  | 2021 | 13 | 6 | 5.6 | 0.65 |
|  | 2022 | 12 | 6 | 5.4 | 0.793 |
|  | 2023 | 5 | 6 | 5.6 | 0.894 |
| Challenge | 2019 | 8 | 6 | 5.2 | 1.165 |
|  | 2020 | 14 | 5.5 | 5.4 | 0.745 |
|  | 2021 | 13 | 6 | 5.2 | 1.166 |
|  | 2022 | 12 | 6 | 5.4 | 0.9 |
|  | 2023 | 5 | 6 | 5.8 | 0.447 |
| Constructive | 2019 | 9 | 6 | 5.4 | 0.726 |
|  | 2020 | 14 | 6 | 5.4 | 0.929 |
|  | 2021 | 13 | 6 | 5.6 | 0.65 |
|  | 2022 | 12 | 6 | 5.3 | 1.155 |
|  | 2023 | 6 | 6 | 5.7 | 0.816 |
| Expertise | 2019 | 9 | 5 | 5.1 | 0.928 |
|  | 2020 | 15 | 5 | 5.2 | 0.775 |
|  | 2021 | 13 | 5 | 5.2 | 0.927 |
|  | 2022 | 12 | 5.5 | 5.2 | 0.866 |
|  | 2023 | 6 | 6 | 5.5 | 0.837 |
| Guidance | 2019 | 9 | 6 | 5.2 | 1.093 |
|  | 2020 | 13 | 6 | 5.5 | 0.66 |
|  | 2021 | 13 | 6 | 5.2 | 1.345 |
|  | 2022 | 12 | 6 | 5.5 | 0.674 |
|  | 2023 | 4 | 6 | 6 | 0 |
| Integrity | 2019 | 9 | 6 | 5.4 | 0.726 |
|  | 2020 | 15 | 6 | 5.7 | 0.458 |
|  | 2021 | 13 | 6 | 5.5 | 0.877 |
|  | 2022 | 12 | 6 | 5.7 | 0.492 |
|  | 2023 | 6 | 6 | 5.5 | 0.837 |
| Resources | 2019 | 9 | 6 | 5.6 | 0.726 |
|  | 2020 | 15 | 5 | 5.3 | 0.816 |
|  | 2021 | 13 | 6 | 5.4 | 0.961 |
|  | 2022 | 12 | 6 | 5.5 | 0.905 |
|  | 2023 | 4 | 6 | 6 | 0 |
| Responsive | 2019 | 9 | 6 | 5.3 | 0.866 |
|  | 2020 | 15 | 6 | 5.4 | 1.056 |
|  | 2021 | 13 | 6 | 5.5 | 0.66 |
|  | 2022 | 12 | 5.5 | 5.2 | 0.965 |
|  | 2023 | 5 | 6 | 5.8 | 0.447 |
| Supportive | 2019 | 9 | 6 | 5.4 | 0.726 |
|  | 2020 | 15 | 6 | 5.7 | 0.488 |
|  | 2021 | 13 | 6 | 5.5 | 0.967 |
|  | 2022 | 12 | 6 | 5.5 | 0.674 |
|  | 2023 | 5 | 6 | 5.8 | 0.447 |
