# Supplementary material for: Preparing the next generation of Complex Networks and Systems scientists: Evaluation results for the Complex Networks and Systems NSF research training program at Indiana University
Source: PLoS One. 2026 Jan 27;21(1):e0334779. doi: 10.1371/journal.pone.0334779 (PMC12843521; doi:10.1371/journal.pone.0334779)
Supplement: S2 File — Table A. CNS NRT doctoral fellows’ average “level of agreement” that they are satisfied with the program and their progress towards their doctoral degree (2019–2023). Table B. CNS NRT Doctoral Fellows’ average “level of agreement” that the CNS NRT program has positively impacted their research skills (2019–2023). Table C. Faculty members’ average “level of agreement” that mentorship has positively impacted doctoral fellows’ research skills (2020–2023). Table D. Doctoral Fellows’ average level of agreement that mentorship has positively impacted their research skills (2019–2023). Table E. Doctoral Fellows’ average level of agreement on mentorship qualities (2020–2023). (DOCX) [file pone.0334779.s004.docx]

**S2 Supporting information. Complex Network Science National Research Trainee (CNS NRT) Annual survey response (2017-2024) descriptive statistics tables.**

**Table A. CNS NRT doctoral fellows' average "level of agreement" that they are satisfied with the program and their progress towards their doctoral degree (2019-2023).**

| **Item** | **Year** | **Responses** | **Median** | **Mean** | **SD** |
| --- | --- | --- | --- | --- | --- |
| Milestone Progress | 2019 | 9 | 5 | 4.8 | 1.093 |
|  | 2020 | 15 | 5 | 4.5 | 1.356 |
|  | 2021 | 15 | 4 | 4.6 | 0.737 |
|  | 2022 | 13 | 5 | 4.7 | 1.032 |
|  | 2023 | 7 | 5 | 4.3 | 1.254 |
| CNS PhD Program | 2019 | 10 | 4.5 | 4.1 | 1.37 |
|  | 2020 | 18 | 5 | 4.7 | 1.018 |
|  | 2021 | 15 | 4 | 4.3 | 1.345 |
|  | 2022 | 13 | 5 | 4.9 | 1.038 |
|  | 2023 | 6 | 5 | 5 | 0.894 |
| 2nd PhD Program | 2019 | 10 | 5 | 4.4 | 1.43 |
|  | 2020 | 17 | 6 | 5.5 | 0.874 |
|  | 2021 | 15 | 5 | 5.4 | 0.632 |
|  | 2022 | 13 | 5 | 5.2 | 0.725 |
|  | 2023 | 7 | 5 | 5.1 | 0.69 |
| Community | 2019 | 10 | 5.5 | 5 | 1.414 |
|  | 2020 | 18 | 6 | 5.6 | 0.705 |
|  | 2021 | 15 | 5 | 4.9 | 1.163 |
|  | 2022 | 13 | 5 | 4.2 | 1.363 |
|  | 2023 | 6 | 4.5 | 4.5 | 0.548 |
| Mentoring | 2019 | 10 | 4 | 4 | 1.155 |
|  | 2020 | 18 | 5 | 5.1 | 0.9 |
|  | 2021 | 15 | 5 | 4.5 | 1.187 |
|  | 2022 | 13 | 5 | 4.7 | 0.63 |
|  | 2023 | 6 | 5.5 | 5.3 | 0.816 |

**Table B.** **CNS NRT Doctoral Fellows' average "level of agreement" that the CNS NRT program has positively impacted their research skills (2019-2023).**

| **Item** | **Year** | **Responses** | **Median** | **Mean** | **SD** |
| --- | --- | --- | --- | --- | --- |
| Writing | 2019 | 9 | 4 | 3.4 | 0.882 |
|  | 2020 | 15 | 4 | 4.2 | 1.146 |
|  | 2021 | 15 | 4 | 3.8 | 1.207 |
|  | 2022 | 13 | 4 | 3.6 | 1.325 |
|  | 2023 | 6 | 4.5 | 4.3 | 1.366 |
| Presentations | 2019 | 10 | 4 | 4.1 | 0.876 |
|  | 2020 | 14 | 5 | 4.6 | 1.342 |
|  | 2021 | 15 | 4 | 4.3 | 1.113 |
|  | 2022 | 13 | 5 | 4.3 | 1.316 |
|  | 2023 | 6 | 5 | 4.8 | 0.753 |
| Technical Skills | 2019 | 10 | 4.5 | 4 | 1.563 |
|  | 2020 | 16 | 4.5 | 4.7 | 1.401 |
|  | 2021 | 14 | 5 | 4.5 | 1.454 |
|  | 2022 | 12 | 5 | 4.9 | 0.793 |
|  | 2023 | 6 | 4.5 | 4.7 | 0.816 |
| Grant Writing | 2019 | 9 | 4 | 3.3 | 1.414 |
|  | 2020 | 11 | 4 | 3.5 | 1.695 |
|  | 2021 | 11 | 4 | 3.2 | 1.168 |
|  | 2022 | 11 | 4 | 3.5 | 1.036 |
|  | 2023 | 5 | 3 | 3.2 | 1.483 |
| Networking | 2019 | 9 | 4 | 3.9 | 0.928 |
|  | 2020 | 17 | 5 | 4.9 | 1.088 |
|  | 2021 | 15 | 4 | 4.5 | 1.125 |
|  | 2022 | 13 | 5 | 4.5 | 1.33 |
|  | 2023 | 6 | 4.5 | 4.7 | 0.816 |

**Table C. Faculty members' average "level of agreement" that mentorship has positively impacted doctoral fellows' research skills (2020-2023).**

| **Item** | **Responses** | **Median** | **Mean** | **SD** |
| --- | --- | --- | --- | --- |
| Grants | 14 | 5 | 4.9 | 1.406 |
| Presentations | 22 | 5 | 5.4 | 0.581 |
| Publications | 20 | 5 | 5.2 | 0.786 |
| Research | 23 | 6 | 5.7 | 0.559 |

**Table D.** **Doctoral Fellows' average level of agreement that mentorship has positively impacted their research skills (2019-2023).**

| **Item** | **Responses** | **Median** | **Mean** | **SD** |
| --- | --- | --- | --- | --- |
| Research | 53 | 6 | 5.3 | 0.95 |
| Publications | 45 | 5 | 5.1 | 0.89 |
| Presentations | 51 | 5 | 4.9 | 1.12 |
| Interdisciplinary | 55 | 6 | 5.3 | 1.03 |
| Grants | 38 | 5 | 4.8 | 1.17 |
| Collaboration | 53 | 5 | 5.2 | 0.98 |

**Table E. Doctoral Fellows' average level of agreement on mentorship qualities (2020-2023).**

| **Item** | **Responses** | **Median** | **Mean** | **SD** |
| --- | --- | --- | --- | --- |
| Accessibility | 53 | 6 | 5.3 | 0.83 |
| Acknowledgment | 45 | 6 | 5.6 | 0.75 |
| Approach | 54 | 6 | 5.5 | 0.69 |
| Challenge | 52 | 6 | 5.4 | 0.93 |
| Constructive | 54 | 6 | 5.5 | 0.86 |
| Expertise | 55 | 5 | 5.2 | 0.84 |
| Guidance | 51 | 6 | 5.4 | 0.94 |
| Integrity | 55 | 6 | 5.6 | 0.66 |
| Resources | 53 | 6 | 5.5 | 0.82 |
| Responsive | 54 | 6 | 5.4 | 0.86 |
| Supportive | 54 | 6 | 5.6 | 0.69 |
